# Developing Time Management Competencies for First-Year College Students Through Experiential Learning: Design-Based Research

**DOI:** 10.3390/bs16010027

**Published:** 2025-12-22

**Authors:** Kunyu Wang, Mingzhang Zuo, Xiaotang Zhou, Yunhan Wang, Pengxuan Tang, Heng Luo

**Affiliations:** Faculty of Artificial Intelligence in Education, Central China Normal University, Wuhan 430079, China; mzzuo@mail.ccnu.edu.cn (M.Z.); zhouxt2023@mails.ccnu.edu.cn (X.Z.); wang_yunhan2022@163.com (Y.W.); pxtang@mails.ccnu.edu.cn (P.T.)

**Keywords:** time management competencies, first-year college students, experiential learning, design-based research

## Abstract

Time management is a critical competency for first-year college students, yet many struggle with limited self-regulation, and existing interventions are often short-term and weakly grounded in theory. This study explored how a design-based research (DBR) approach integrating experiential learning and digital tools could strengthen students’ time management skills. From 2021 to 2023, 238 first-year students at a research university in central China participated in a three-month hybrid Freshman Orientation Seminar, with data collected from daily submissions via a WeChat mini-program. Over three iterative DBR cycles, the intervention combined experiential learning theory with authentic time management practice, guided by quantitative and qualitative evidence to refine the pedagogical model. The process yielded six design principles and a supporting digital tool. In the final iteration, students demonstrated substantial gains, including improved planning, greater task completion, more accurate time allocation, and higher satisfaction with time use. These findings suggest that sustained, theory-guided experiential learning, when supported by digital tools, can significantly enhance time management competencies. The study contributes practical strategies for embedding self-regulated learning into higher education through technology-enhanced experiential approaches.

## 1. Introduction

The transition from secondary education to competency-based undergraduate programs demands heightened autonomy, as students must balance academic and extracurricular tasks while under time pressure (Gore & Metz, 2017; Shaunessy-Dedrick et al., 2015). Educational research has increasingly examined time management through a self-regulated learning (SRL) perspective, in which it is viewed as an important regulatory competence associated with students’ academic engagement and persistence (Britton & Tesser, 1991; Zimmerman, 2013). Within SRL frameworks, time management is widely recognized as a central regulatory competence that enables learners to allocate effort, prioritize tasks, and sustain engagement under time pressure (Wolters & Brady, 2021). Poor time management can lead to a decline in academic performance (Van Sluijs & Matzat, 2023), serious mental health issues (Aeon et al., 2021), and even dropping out of school (Xavier & Meneses, 2022). Chinese secondary education intensifies these challenges through rigid schedules that are controlled by teachers and parents, which limit students’ opportunities to practice time management. This systemic gap leaves students unprepared for the university environment. For those not trained in time management, the effort required to succeed and maintain well-being in college is often quite challenging (Cifuentes Gomez et al., 2022). Taken together, these issues highlight the educational importance of developing effective, evidence-based interventions that can support first-year students’ transition to more autonomous learning environments, in which learners are increasingly expected to take responsibility for regulating their behaviors and time use.

While time management is a trainable competence, existing interventions in higher education face three systemic limitations (Duckworth et al., 2018; Valovirta & Mannevuo, 2022). First, such training is often limited to brief seminars during orientation periods rather than sustained curricula (Arco-Tirado et al., 2011). Second, few institutions provide longitudinal programs to reinforce these skills (Häfner et al., 2014). Third, most current approaches prioritize didactic instruction over experiential learning and lack robust theoretical frameworks to guide implementation (Trentepohl et al., 2022). This contrasts with evidence showing that the higher-order thinking skills essential for self-regulation develop through practical application (Nenzhelele, 2014). Accordingly, recent research has increasingly examined time management from an SRL perspective, viewing it as a regulatory competence that must be developed through sustained engagement in authentic learning contexts rather than through instruction alone (Du et al., 2025). Nevertheless, recent reviews indicate that empirical intervention research remains limited and insufficiently grounded in sustained (Simón-Grábalos et al., 2025), practice-oriented instructional designs.

To address the need for sustained, practice-oriented approaches to developing time management, experiential learning offers a pedagogical framework that emphasizes learning through action, reflection, and iterative refinement in authentic contexts. Experiential learning is a way for students to integrate classroom learning with real-life experiences through reflective practice. This can provide strategic guidance for improving time management competencies (Groves et al., 2013). As the Chinese proverb emphasizes, “true knowledge emerges through practice”. Some higher educational institutions have embraced experiential learning in areas such as entrepreneurship education (Motta & Galina, 2023), occupational skills training (Arjmandi et al., 2023), and subject knowledge (Dobbs-Oates, 2022). Existing research has explored how to develop students’ acquisition of knowledge and skills through the four phases of experiential learning (Liu et al., 2022), but there has been no examination of the appropriate pedagogical strategies for developing time management competency. Current studies and practical applications have also not extensively examined how educators implement experiential learning. As a result, educators often struggle to systematically design activities requiring continuous observation and analysis of student experiences (Bian et al., 2022). However, despite increasing attention to experiential forms of learning, there remains limited guidance on how theoretically informed, practice-oriented designs can be systematically developed to promote time management competence. This gap underscores the need to connect experiential learning theory with concrete instructional design decisions that directly address students’ time management challenges.

To solve the practical problems of guiding the development of time management skills among first-year university students through experiential learning, this study employed a design-based research (DBR) approach. By linking experiential learning theory with iterative design cycles, this research aims to generate actionable principles that respond to both theoretical expectations and classroom realities. More specifically, we sought to answer the following research questions:

(1) How can experiential learning theory be designed and implemented?
(2) What design principles are valued by the students and why?
(3) What design principles are not valued by the students, and what are the possible ways to improve them?
(4) What are the benefits and limitations of this experiential learning approach?

## 2. Theoretical Framework for Design

The theoretical framework for designing experiential learning activities to cultivate time management competencies was informed by theories related to time management and experiential learning, with selective reference to self-regulated learning (SRL).

### 2.1. Time Management

There is no consensus on the major components of time management. Theorists typically depict it as a multidimensional process that encompasses planning, monitoring the use of time, reflecting, adjustments, and deliberately structuring or allocating the use of time (Britton & Tesser, 1991; Macan et al., 1990; Xavier & Meneses, 2022). Individual experience and reflective adjustment are also considered to be essential elements in time management (Khiat, 2022). It is generally believed that individuals with better time management exhibit positive psychological tendencies and behavioral characteristics in related dimensions (Abrantes et al., 2018).

Empirical studies have demonstrated that effective time management significantly benefits college students, particularly first-year undergraduates, by improving academic performance, sleep quality, emotional regulation, and subjective well-being (Kuzilek et al., 2023). The transition from high school to college considerably increases the academic load (Kahu & Picton, 2022). The college curriculum tends to be more complex and recondite than high school, thus requiring increased dedication and hard work. Proactive time allocation reduces anxiety and enhances achievement through structured task completion (Ribeiro et al., 2019). 

At the same time, students must balance academic responsibilities with social and personal commitments in their new autonomous environment (Sotardi & Dutton, 2022). These challenges contribute to first-year attrition rates globally, which positions time management as a critical intervention strategy for improving education outcomes (Kutija & Skrbinjek, 2024). However, most research has focused primarily on cross-sectional studies exploring the relationship between time management and other variables, such as boredom, motivation, academic performance, parental autonomy support, and self-control, by distributing questionnaires or scales (Xie et al., 2020), which limits the exploration of strategies for nurturing education.

The training programs that exist to build time management competencies tend to be quite similar, with a focus on a few key areas: prioritizing tasks, setting goals, making daily plans, and tracking progress (Bargmann & Kauffeld, 2023). Teaching knowledge or practicing strategies through courses is now the mainstay of such training. Courses that combine time and effort management with acceptance and commitment therapy have been shown to help students enhance their ability to manage both their time and their efforts effectively (Hailikari et al., 2021). However, the specifics of the curriculum and the production of specific effects remain a black box for teachers and students who want to improve. Empirical studies have confirmed that practical skill-building surpasses theoretical knowledge transmission, but most implementations lack robust theoretical frameworks for cross-contextual application (Trentepohl et al., 2022). Although recent scholarship has explored time management through diverse lenses, determining how to integrate these theories into practical application effectively remains a challenge.

### 2.2. Experiential Learning

Experiential learning is the process of creating knowledge through the transformation of experience (D. A. Kolb, 1984). If they are to be effective, learners need four different kinds of abilities, which are related to concrete experience, reflective observation, abstract conceptualization, and active experimentation. This involves a continuous cycle-forward process in experiential learning, which enhances higher-order thinking skills while boosting student motivation and learning outcomes (Tembrevilla et al., 2024). Growing empirical evidence and institutional confirmation support the critical role of experiential learning in fostering academic success through deep, engaged learning (Jonathan & Laik, 2024).

Researchers have contributed a wide spectrum of interpretations and models on the design and theoretical underpinnings of the four stages of experiential learning. An early study emphasized that the focus of experiential learning should be on the concrete experiential stage (Joplin, 1981). More recent work, however, suggests reflective thinking and abstract generalization may be more important (Falloon, 2019), with reflection possibly constituting the most crucial element of experiential learning, alongside the individualization of concrete experience and tutors (Jacob & Boyter, 2023). Analyzing the significance of the various stages of experiential learning on a case-by-case basis is also thought to be essential (Taneja et al., 2023). Existing research thus does not provide a clear view of the importance of the different phases and little discussion of the design and implementation of the active experimentation phase.

Experiential learning theory is primarily applied at present in fields such as medicine (Goldman et al., 2020), business (Perusso et al., 2020), and teacher education (Scarparolo & Mayne, 2022), which emphasize the combination of theory and practice to cultivate professional ability. Time management competencies can also be improved in experiential learning, even if the goal is to develop other professional skills (Brown et al., 2020). Through practice and practical application, learners can gain a deeper understanding of time management principles and develop relevant skills. This approach to learning can also stimulate learners’ interest and motivation, thus making them more focused and engaged (A. Y. Kolb & Kolb, 2009).

### 2.3. Integrating Self-Regulation, Time Management, and Technology Support

Building on the conceptualization of time management as a developmental competence and experiential learning as the primary pedagogical approach, this study adopts self-regulated learning (SRL) as an integrative theoretical framework to explicate how time management develops through learners’ regulatory processes over time. In contrast to views that treat time management as a discrete or purely behavioral skill, SRL conceptualizes it as a form of temporal regulation embedded within broader cycles of self-regulation, including planning, monitoring, effort regulation, and reflective adaptation (Zimmerman, 2002; Pintrich, 2000). Within established SRL models, regulatory behaviors such as scheduling tasks, allocating time, monitoring task progress, and adjusting effort are consistently identified as core mechanisms through which learners coordinate their learning activities across temporal demands (Beishuizen & Steffens, 2011; Fayaza & Ahangama, 2024). Accordingly, prior research has increasingly positioned time management as a central self-regulatory competence rather than a peripheral study skill, highlighting its close associations with academic engagement, persistence, and effective learning strategies among university students (Tao & Hanif, 2025; Wolters & Brady, 2021). From this perspective, time management competence is understood to emerge through repeated engagement in regulatory cycles rather than through one-time instruction. This SRL-based interpretation provides a coherent theoretical foundation for integrating time management development with experiential learning designs that emphasize iterative practice, feedback, and behavioral adjustment over time.

Within an SRL framework, technology plays a critical role in supporting time management development by externalizing and scaffolding learners’ regulatory processes. A growing body of research in technology-enhanced learning demonstrates that digital tools can support key SRL components by enabling learners to articulate plans, visualize workload distribution, monitor progress, and engage in data-informed reflection (Panadero, 2017; Xu et al., 2023). Learning-analytics dashboards, time-tracking systems, and automated feedback mechanisms provide timely information that helps learners monitor and adjust their time-use behavior (Lim et al., 2023; Matcha et al., 2019; Paulsen & Lindsay, 2024). Importantly, by capturing behavioral traces of learners’ planning, monitoring, and time allocation, technology not only supports learners’ self-regulation but also provides instructors with empirical evidence for examining how time management practices evolve over time. In this way, technology functions as a critical mediating component that connects SRL theory with experiential learning design and the assessment of time management development. In experiential-learning contexts, technology has also been shown to deepen engagement with concrete experience, reflection, and experimentation by making learning processes more visible and by facilitating iterative evaluation (Rigopouli et al., 2025; Jin & Sercu, 2025). These studies highlight that technology can work in tandem with experiential learning to structure reflective opportunities, capture behavioral evidence, and support strategy refinement.

In this study, time management is the target competency, understood through the lens of SRL as a temporal regulation process. Experiential learning provides the pedagogical approach that enables students to practice and refine this competency through iterative cycles of concrete experience, reflection, conceptualization, and active experimentation (D. A. Kolb, 1984; Tembrevilla et al., 2024). Technology functions as both a pedagogical support and an assessment tool: it facilitates students’ engagement in experiential-learning activities by providing real-time planning, monitoring, and feedback mechanisms, while simultaneously generating behavioral data that allow for the evaluation of changes in time-management practices over time. Taken together, SRL theory, experiential learning, and technology form an integrated framework that underpins the design of the intervention and the formulation of its design principles.

### 2.4. Theoretical Assumptions for the Design Phase

The design principles and decisions for developing time-management competencies build directly on the integrated framework outlined in Section 2.3, which connects self-regulated learning, experiential learning cycles, and technology-supported monitoring. Based on this framework and the literature, the principles derived from the four dimensions of experiential learning theory are summarized in Table 1.

Design Principles D1 and D2 emphasize task-driven, authentic experiences to promote engagement by embedding time management tasks in real-life contexts. This approach aligns with research advocating the contextualization of abstract skills to enhance motivation and facilitate knowledge transfer. By requiring students to align academic tasks with their existing routines, the intervention activates situated cognition (Hsu et al., 2023). D3 and D4 enrich the reflective observation phase by embedding scaffolding and self-monitoring, which facilitate reflective processes and foster metacognitive awareness through data-informed reflections (Zimmerman, 2013). Structured scaffolding for recording, monitoring, and reflecting helps students externalize and clarify their time use, revealing gaps between actual behavior and perceived productivity. D5 and D6 extend the experiential learning cycle by enabling teamwork for communication and interaction (Bhute et al., 2022) and by supporting iterative practice for time management skill development and sustained behavioral change. Overall, students shifted from passive completion to active implementation in their time management during the experiential learning activities. This synergy generated an iterative learning process characterized by cognitive dissonance, strategy visualization, behavioral validation, and cognitive refinement, thereby fostering continuous development. Abstract strategies were transformed into specific actions through practical design (Podolskiy, 2014).

## 3. Methods

### 3.1. Design-Based Research

DBR employs iterative cycles of educational improvement through collaborative real-world implementation to generate contextually grounded design principles. This methodology systematically evaluates instructional design frameworks using mixed-methods analysis to bridge theoretical refinement and practical application (Spector et al., 2014). Our study adopted DBR to provide an empirical evaluation of experiential learning design principles for enhancing undergraduate time management competencies. Through three iterative cycles of design, implementation, and evaluation, we collected multi-modal data to assess the effectiveness of the framework and inform theoretical advances.

### 3.2. Research Context

This study took place at a research university in central China over the span of 3 years. The target course, the Freshman Orientation Seminar, was designed for first-year students and delivered through a hybrid model combining online and offline smart classrooms. The course aimed to support students’ transition to university and enhance their time management skills. From 329 initial participants across 3 cohorts, 238 educational technology and digital media majors (aged 17–20) completed the full 3-month intervention with ≤25% missing daily data submissions. The gender distribution was roughly 68.1% female to 31.9% male. Only about 14.7% of the students had prior experience with time management. Nearly 40.8% had attempted to manage their time but found maintaining the practice difficult over the long term. Just over a third (36.1%) of the students had heard of time management from others but had not tried it themselves.

This study was conducted following the ethical standards of the Helsinki Declaration. The research protocol and instruments were reviewed and approved by the author’s Institutional Review Board. All participants provided their written informed consent to participate in this study. They were informed that participation was voluntary and that their personal information would remain confidential in any publications or presentations.

### 3.3. Instructional Process

Across the semester, the twelve weekly sessions collectively formed a complete experiential learning cycle, within which multiple smaller cycles were embedded. Each smaller cycle consisted of two consecutive seminars and the fourteen days of technology-supported out-of-class activity tracking between them, guiding students through concrete experience, reflective observation, abstract conceptualization, and active experimentation.

**Figure 1:**
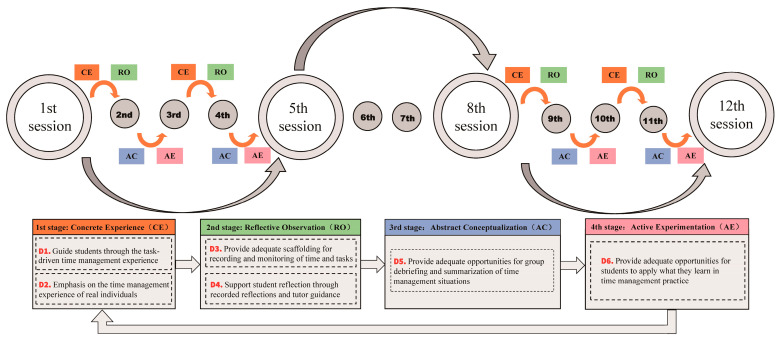
Developing Time Management Competencies through Experiential Learning Model.

To structure the intervention consistently across years, each iteration commenced in late September with the introduction of time management strategies, semester plans, and course schedules (Figure 1). This was accompanied by orientation activities and phased task releases (D1 and D2). From October, the intervention progressed through three stages. A 90-min offline seminar took place once a week at all stages. In the first seminar of each cycle, instructors introduced tasks and provided explanations that guided students into the experiential learning process, particularly the concrete experience and reflective observation phases. In Stage 1, students completed daily 15-min tasks to log activities, assess effectiveness, and reflect on their experiences (D2 and D3). During the second seminar of each cycle, instructors reviewed students’ recorded data and offered feedback to support abstract conceptualization, enabling students to identify patterns and refine strategies. Weekly reflections summarized time use, identified issues, and proposed improvements, with teacher feedback provided in the following class (D4). At the end of the month, students presented a group or individual summary report (D5). Stage 2 (November) functioned as a control phase, during which task completion was not mandatory. In Stage 3 (December), all activities from Stage 1 were resumed to re-engage students in active experimentation (D6). Across Stages 1 and 3, four such small experiential learning cycles were completed, providing repeated opportunities for practice and refinement.

### 3.4. Data Collection and Analysis

A multi-source approach was used to evaluate the instructional design, incorporating classroom observations, student daily records, questionnaires, and interviews. All 12 sessions were recorded with rear-mounted cameras in the smart classroom, supplemented by on-site observations and field notes from teaching assistants and researchers, capturing key aspects such as student reports, teacher feedback, and scaffolding strategies. Student daily records, generated through embedded scaffolds in experiential learning activities, served both as an intervention and a data collection tool to assess changes in time management. As the measures consisted of student-recorded behavioral logs rather than psychometric scales, instrument quality was addressed through design-based refinement rather than internal consistency testing; the digital tool was iteratively revised across the three DBR cycles based on expert feedback and student interviews. Semi-structured interviews were conducted after each iteration to capture students’ experiences, while the demographic questionnaire was administered prior to the intervention to collect factual background information. To improve transparency, the Supplementary Materials includes the questionnaire, interview protocols, and the digital tool’s interface and data fields. In addition, the analysis data are publicly available in a third-party repository.

For iteration-level comparisons, a one-month segment of continuous behavioral records was extracted from each cycle. Indicators such as recording days, completed tasks, and daily time allocation were calculated. Non-parametric tests were used due to non-normality (Kolmogorov–Smirnov, *p* < 0.001). Quantitative analyses were conducted in SPSS 27, and qualitative data were thematically coded in NVivo 11.

## 4. Results

The scope of this study covered three cohorts of first-year students over three consecutive years, leaving 238 students at the end of the study after removing insufficient participation and too much missing data. We first present quantitative data comparisons demonstrating model improvements across three iterations, then detail specific improvements and their rationale iteration by iteration.

### 4.1. Iterative Evolution of Time Management Competencies

Across the three iterations, the intervention maintained a consistent set of daily behavior indicators while progressively upgrading the data collection formats. As summarized in Table 2, students’ time management behaviors were recorded using a spreadsheet-based log in the first iteration, an online questionnaire in the second iteration, and a mobile mini-program with real-time logging and feedback in the third iteration. This iterative evolution aimed to improve data granularity and recording continuity while ensuring the comparability of core indicators.

Based on the data collected across these iterations, Table 3 shows significant improvements in students’ time management competencies across three iterations. Following the three core processes of self-regulated learning (planning, performance, and reflection) we evaluated students’ time-management development across four dimensions: planning behaviors, execution and monitoring, time allocation, and self-reflection, operationalized through eight specific indicators. Measured variables include days of recording, total planned and completed tasks, diversity of completed categories, planned completion rate, average daily time on independent learning and extracurricular activities, and daily self-evaluation scores. The detailed data collection form specifying the daily behavioral indicators is provided in Supplementary Materials S1.3. Because these indicators were not normally distributed, non-parametric analyses (Kruskal–Wallis H) were conducted using data from the final month of each iteration. 

Across the behavioral measures, effect sizes were consistently large (*η*^2^ = 0.139–0.447), indicating strong practical significance beyond statistical significance (Richardson, 2011). The number of recording days increased by 86.7% (*η*^2^ = 0.447), reflecting markedly improved adherence to daily time-management routines. Compared with the first and second iterations, the third showed more than a twofold increase in both total planned and completed tasks with only a slight decline in completion rate, demonstrating improved time management capacity. Significant differences across iterations indicate evolving time allocation patterns. Although independent learning time initially declined, this likely reflects better adaptation and more realistic self-regulation, and the steady increase in extracurricular time suggests the development of a more balanced, sustainable routine. Daily self-evaluation reflects students’ moment-to-moment judgments of how well they managed their learning and tasks each day. The significant increase (*η*^2^ = 0.421) across iterations indicates that students became more intentional and accurate in monitoring their daily behaviors.

### 4.2. First Iteration

Our experiential learning activities on time management ran from September to December 2021 with 109 first-year students, with 71 remaining actively engaged by the end of the semester.

This iteration addressed two research questions:

RQ1.1: How do first-year students participate in and respond to the experiential learning activities in the initial design?

RQ1.2: What challenges emerge when technology-supported experiential learning activities are introduced to support time management?

#### 4.2.1. Implementation

Guided by six experiential learning design principles, instructors conducted 12 workshops. The first session introduced core time management concepts, tools, and the online platform. Through structured tasks, students documented and analyzed their routine behaviors, engaged in problem-solving in small groups, and participated in weekly reflection and peer sharing. Supplementary Materials Figure S1 illustrates the scaffolded tool that supported students’ engagement in the experiential learning cycle by guiding them through planning, performance, and reflection activities. This tool not only facilitated students’ participation in the experiential learning process but also served as the primary source of behavioral data used to evaluate improvements in time management across iterations. These tools were developed based on the experiential learning design principles, particularly D3 and D4. Faculty support was sustained through October before supervision was reduced to encourage autonomy. In December, students entered the active experimentation phase, the primary period for assessing time management improvements.

#### 4.2.2. Instrumentation

In the first iteration, four types of data were collected to assess students’ time management development: classroom observations, student daily records, semi-structured interviews, and a baseline questionnaire. Twelve students were selected based on classroom performance, divided into high- and low-performing groups, and each interview lasted 30–40 min. Table 4 summarizes the data categories, variables, and instruments used in this iteration.

The structure and categories of the daily-record tool were initially informed by established time-management practice frameworks and refined through several rounds of consultation with four domain experts during the preparation phase. Classroom observations, field notes, and interview transcripts were thematically coded in NVivo 11 based on the design principles and on identified strengths and weaknesses to guide refinements for the next iteration.

Student daily records, completed through the embedded scaffolds in the experiential learning activities, were coded, categorized, and analyzed as behavioral log data to capture changes in time management behaviors. Planned task checklists were cross-referenced with timeline-based activity logs (Supplementary Materials Figure S1) to identify completed, uncompleted, and unplanned tasks. All tasks were coded into 9 predefined categories (e.g., coursework, reading); for example, “Finish math work” as coursework and “Jogging” as physical exercise. Variables such as task duration, completion rating, and self-evaluation scores were extracted and standardized. Eight behavioral indicators were computed using frequencies (e.g., days of recording), totals (e.g., total completed), and daily averages (e.g., daily independent learning time) for iteration-level comparisons (see Table 3). Daily independent learning time is the average time per day on academic-related activities (coursework, additional Learning, professional practice, reading), while daily extracurricular time is the average time on non-academic activities (physical exercise, hobbies and interests, social activities, electronic use). The selection of these eight indicators followed the forethought, performance, and reflection phases of self-regulated learning (SRL), which guided how time-management competencies were operationalized across the three iterations.

#### 4.2.3. Evaluation

This study analyzed first-year students’ perceptions of Kolb’s experiential learning phases for the time management program through integrated questionnaires, interviews, observations, and behavioral logs to reveal their attitudes toward the intervention (Table 5).

Student opinions on the concrete experience phase of the initial design varied, especially regarding the mandatory tasks. Most students said, “I lack the motivation to start without mandatory tasks.” Some valued external motivation, while others saw time management as inherently self-driven. One student commented, “Time management is about self-awareness; mandatory tasks leave me exhausted and make wasted time seem useless.” However, most students recognized the value of tracking real practice data to clarify misconceptions about time use. One student noted, “I thought I was busy with many things, but after reviewing the actual situation, I realized it wasn’t true.”

In the reflective observation phase, students recognized the benefits of structured self-reflection on time management. They described classes as a “spiritual awakening” at the week’s start, which motivated ongoing self-improvement reflections. Structured tools like Excel templates helped identify gaps in their routine, such as increasing exercise duration and maintaining a regular sleep schedule. However, they found documenting repetitive details to be time-consuming and burdensome. While valuing reflection for self-improvement, some noted that academic assessments endangered authenticity, as the pressure to impress might lead to embellishment. Students want to protect their privacy while still enabling the sharing of experiences.

In the abstract generalization phase, students preferred independent work, although group dynamics critically influenced outcomes. Proactive groups achieved deeper analysis through mutual oversight (“identifying root causes collaboratively”), while disengaged groups triggered a broken window effect with superficial record compilation. Only two groups demonstrated effective collaboration.

To support the active experimentation phase, a dedicated period in November allowed students to reflect independently on their experiences, identify challenges, and plan improvements. This phase did not include centralized teacher feedback and mandatory daily tasks. The self-directed phase in November reduced resistance to documentation by December. Students then reported clearer self-awareness. Most students summarized this change: “Upon pausing, I realized the importance of timing for myself, and felt I performed better in the last month.” Students cyclically adjusted their routines through daily logging and weekly summaries, evaluated their time allocation, identified issues, and planned improvements for the upcoming week.

Overall, the feedback collected across the four experiential learning phases revealed consistent challenges related to task load, documentation burden, and uneven peer participation. These patterns suggested a need to adjust the balance between structure and autonomy, enhance the clarity and efficiency of the recording tools, and strengthen supports for collaborative analysis.

#### 4.2.4. Reflection on Principles and Decisions

A review of the six key principles of our model showed that most aligned well with the intended educational objectives. By engaging students in required tasks, the model introduced an experiential learning cycle that encouraged students to identify and address personal challenges, reflect on experiences, and implement improvements. This process supported the development of student agency, enhanced time management skills, and built self-confidence.

However, some principles required further refinement to improve their focus and effectiveness. Principle D3, for example, did not provide enough guidance on addressing core time management issues, and its use was limited by the burden of documentation and concerns about privacy. Principle D4 needed to be restructured to incorporate timely, actionable feedback through a dual mechanism: instructor evaluations and structured self-reflection to support learners in recognizing progress and addressing gaps. For Principle D5, the focus needed to shift from general collaboration to balancing group interaction with individual accountability, thus ensuring that both collective and personal learning needs are addressed.

#### 4.2.5. Revision

Based on the findings gathered in the evaluation and reflection phase, several adjustments were made to refine the instructional design. Feedback regarding the burden of early mandatory tasks and repetitive documentation led to streamlining the recording procedures and adjusting the balance between structured requirements and learner autonomy. Observations of uneven group engagement resulted in modifications to peer-support structures, particularly in activities requiring collective analysis. These insights also indicated the need to further refine specific components of the experiential learning design. Accordingly, in the subsequent iteration, these findings informed targeted refinements to the corresponding design principles, especially those related to reducing cognitive load and improving the efficiency of time-management documentation.

First, the time-recording scaffolding was optimized by migrating from Excel to the Sojump web platform and establishing 9 standardized activity categories (e.g., academic, extracurricular). This dual modification simplified data entry procedures while enhancing privacy through segmented data storage. This simultaneously addressed students’ surveillance concerns and improved record authenticity. The standardized format also enabled educators to assess time management skills consistently through unified evaluation protocols.

Second, we developed a tiered feedback system combining structured reflection with differentiated guidance. In the next iteration, students would participate in weekly group sessions analyzing time management patterns through five core dimensions: activity planning, unplanned event adaptation, duration measurement, completion evaluation, and effectiveness assessment. Instructors would deliver class-wide guidance informed by performance trends and contextual factors. This multilevel approach aligns macro-level curricular objectives with micro-level student needs through integrated standardized metrics and personalized support (Lehmann et al., 2014).

Third, the group reporting framework was enhanced through individualized tracking components (Francis et al., 2020). In the next iteration, students would submit detailed weekly plans every Monday and comprehensive progress reports each Friday, documenting challenges, efforts, and achievements in time management. This personal accountability mechanism was intended to supplement rather than replace group collaboration elements. Additionally, students would systematically track sleep patterns, study situations, extracurricular activities, and emotional states through daily summative assessments to create integrated datasets for holistic self-evaluation.

Collectively, these revisions established a balanced ecosystem combining technological streamlining (web platform), structured reflection (multilevel feedback), and personalized tracking (individual planning). The revised system maintained group collaboration value while strengthening self-regulation capabilities through standardized processes that reduce administrative burdens, ensure assessment consistency, and enable a nuanced understanding of students’ time management development.

### 4.3. Second Iteration

Our second iteration ran from September to December 2022 with 110 first-year students, adhering to the six design principles. Compared to the first iteration, three principles for the two experiential learning phases were modified. By the end of the semester, 65 students remained actively engaged.

Building on the first iteration, which identified the need to strengthen reflective observation and performance evaluation processes (D3–D5), the second design cycle focused on the refinement and technological enhancement of self-monitoring and reflection tools. This iteration was guided by the following research questions:

RQ2.1: How effective are the revised monitoring and reflection scaffolds in supporting students’ analysis, regulation, and adjustment of their time-use behaviors?

RQ2.2: To what extent does the mobile-based implementation of these scaffolds improve students’ engagement, consistency of logging, and accuracy of behavioral data?

RQ2.3: How does the integration of group-level or collective feedback influence students’ awareness of inefficiencies and their subsequent strategy adjustments?

#### 4.3.1. Implementation

In this implementation, we not only improved the basic design principles but also reworked the tool (Supplementary Materials Figure S2): (i) replacing manual logs with 15-item online selections spanning biometric data (sleep/emotion), 9 categorized behaviors (e.g., academic/professional/exercise), and four standardized evaluation dimensions (plan, time, completion, quality assessment); and (ii) integrating daily 5-point satisfaction ratings to quantify self-regulated learning patterns. These tools were developed based on the experiential learning design principles, particularly D3 and D4, and allow students to document and reflect on their time-management behaviors directly on mobile devices. They served a dual function in the intervention: providing structured scaffolding that supported students’ engagement in the experiential learning cycle and generating behavioral data used to evaluate and compare the effectiveness of the design principles across iterations. At the end of the week, students receive a personalized report, summarized by the teaching assistant, that highlights key metrics such as task completion, sleep patterns, and individual weekly self-assessments. Group members are then given the autonomy to choose from a range of activities: group discussions, individual reflection, and peer comparison. 

#### 4.3.2. Instrumentation

In the second iteration, data collection and instruments remained consistent with the first, except that Sojump replaced Excel for recording students’ daily time management behaviors through structured digital input, eliminating manual coding. Researchers directly calculated frequencies, totals, and daily averages for behavioral indicators to support cross-iteration comparisons. Interviews were conducted with 12 students selected based on classroom performance.

#### 4.3.3. Evaluation

In this iteration, while the overall implementation of the teaching principles was acceptable, the outcomes fell short of expectations. The implementation revealed persistent gaps between pedagogical design and practical outcomes. The digitized tracking system reduced the time spent on monitoring records by two-thirds and initially engaged students and reflection through streamlined data entry(Table 6). One student noted, “If I find that I don’t have anything to fill out today, I feel guilty and will adjust my behavior the next day.” The actions of the next day were thus influenced by the data from the previous day. However, some limitations also emerged.

First, the tool had several design flaws. Redundant identity verification fields lacked auto-save functionality, which forced repeated manual input. Simultaneously, the input-only interface blocked access to retrospective data, thus preventing students from reviewing past entries. These technical limitations generated operational friction, which disrupted workflow continuity. Students consequently reported diminished capacity for longitudinal self-assessment due to fragmented behavioral data. Many students mentioned in interviews and reflections that, “Although I consciously reflect on my time management when filling out the online link, there’s no place to review it afterward, and I don’t have a good way to summarize my issues and plans for the next week over the weekend.” Second, delayed weekly feedback cycles and non-visualized reports diminished actionable insights, as most groups struggled to translate generic suggestions into behavioral adjustments. Third, ambiguous collaboration objectives and unstable group dynamics led to inconsistent task ownership, although daily logging fostered micro-level awareness, with over half of the participants self-correcting time allocation within three weeks.

#### 4.3.4. Reflection on Principles and Decisions

The second iteration of the experiential learning model revealed both the efficacy and unresolved challenges across three core principles. Post-modification analyses indicated that the restructured D3 scaffolding successfully mitigated time inefficiencies and privacy concerns through scaffolded tasks. Preliminary learner feedback underscored the need for embedded retrospective tools—analogous to Excel’s personal analytics features—to enable sustained self-regulation. Concurrently, the D4 mechanism had critical limitations in terms of timeliness and interpretability, with most of the participants describing feedback as “disconnected from context.”

To address this, the developments for the next iteration prioritized multimodal feedback systems to transform raw metrics into actionable, visually intuitive insights. The D5 (Group Reflection) principle, by fostering collaborative problem-solving, inadvertently diluted individual accountability. Theoretical alignment with self-directed learning paradigms (e.g., Zimmerman’s cyclical phase model) necessitates a redesign emphasizing metacognitive scaffolding—shifting from collective consensus-building to structured frameworks for identifying individual knowledge gaps. 

#### 4.3.5. Revision

Iterative refinements to the experiential learning design principles improved pedagogical efficacy through technological optimization and enhanced learner agency. Improvements streamlined user interactions, optimized data processing, and personalized feedback. In the next iteration, shortcomings from the second round were addressed by refining data input and output. Migrating from Sojump-based tracking to an embedded WeChat mini-program improved accessibility through native platform integration. Automated reminders, editable entries, and cloud synchronization supported compliance and longitudinal self-monitoring without extra downloads. Integrated user profiles removed repetitive demographic inputs, while interruptible task flows accommodated real-world contingencies, aligning with real experience design principles (Kukulska-Hulme, 2021). Feedback mechanisms combined temporal immediacy with social comparison theory (Festinger, 1954). Post-task dashboards visualized personalized metrics alongside cohort averages to contextualize individual performance. 

### 4.4. Third Iteration

The third iteration ran from September to December 2023 with 110 first-year students, with 102 remaining consistently involved by the end of the semester. Compared to the second iteration, three principles for the two experiential learning phases and scaffolding tool were modified.

Building on the refinements from the previous iterations, the third iteration focused on evaluating the effectiveness of technology-supported scaffolds. Specifically, this cycle investigated:

RQ3.1: How the revised time-management monitoring and reflection scaffolds, when implemented through a WeChat mini-program, enhanced students’ engagement and regulatory behaviors.

RQ3.2: How individualized, technology-enabled visual feedback influenced students’ awareness, strategic adjustments, and sustained participation compared with earlier group-based feedback approaches.

RQ3.3: Whether the integrated digital scaffolding system contributed to more consistent and measurable improvements in students’ time-management competencies.

#### 4.4.1. Implementation

The three-month experiential learning activity integrated a WeChat mini-program, Self-Management Assistant, developed by the research team to support time management (Supplementary Materials Figure S3). Students could enter personal data and view visual feedback, including completion items and time distribution, over daily, weekly, or custom periods. The mini-program was also designed for teachers, who could use automated data to identify areas needing adjustment. Class-wide comparisons and average scores further supported peer connection among students. These tools were developed based on the experiential learning design principles, particularly D3, D4 and D5.

#### 4.4.2. Instrumentation

In the third iteration, data collection and analysis followed the established procedures, with the main change being the adoption of a WeChat mini-program for mobile-based daily record input and real-time visual feedback. Exported data were structured, eliminating additional coding, and key behavioral indicators were directly calculated using frequency, totals, and daily averages.

#### 4.4.3. Evaluation

In the third iteration, the implementation of overall teaching principles and results reached a relatively satisfactory state. Students reported high satisfaction with the WeChat mini-program, describing it as “convenient and covers a wide range.” The mini-program improved time management efficiency and enhanced the impact of personalized data feedback. Its objective data presentation increased students’ awareness of change and supported behavior improvement. Students found the feedback more effective and better suited to their needs than teacher guidance. Group cooperation was minimized in this iteration, as students noted that fixed professional class groups were less suitable for personal time management due to privacy concerns.

## 5. Discussion

This study directly connects back to the theoretical framework by illustrating how technology-supported experiential learning collectively shaped the development of students’ time-management competencies. Building on this alignment, evidence from behavioral records, weekly reflections, classroom observations, and student interviews was used to evaluate the implementation of the six design principles across iterations, revealing how each principle contributed to the observed improvements in planning, monitoring, and task execution.

### 5.1. Key Findings

This study used experiential learning to improve first-year university students’ time management skills and support their transition from high school. Effectiveness was examined through three iterative cycles incorporating student input and feedback on design principles. Six design principles (Table 7) were validated. The intervention maintained conventional teaching processes while integrating scaffolded self-monitoring tools (e.g., reflection journals, time-tracking dashboards) into a WeChat mini-program.

The findings across the three design cycles demonstrate consistent and measurable improvements in first-year students’ time-management competencies. Behavioral data showed increases in days of recording, total planned and completed tasks, diversity of completed categories, and students’ daily self-evaluations. These gains reflect greater persistence, enhanced planning behavior, and more stable engagement over time. The results also align with established SRL research indicating that structured monitoring promotes self-regulation and engagement (Won et al., 2025). Although task volume increased substantially, students maintained comparable completion rates, suggesting that appropriate experiential-learning design principles can effectively manage cognitive load and prevent efficiency loss even as task demands grow (Evans et al., 2024).

Students’ qualitative reflections further explain these behavioral changes. Many reported heightened awareness of how small daily activities (mealtimes, commuting, digital browsing) accumulate to reduce productive hours, prompting them to adopt allocation-oriented strategies. This heightened awareness is consistent with findings that self-monitoring tools enhance learners’ ability to evaluate effort and adjust habits (Banihashem et al., 2022). The portable, privacy-sensitive digital tools used in this study increased students’ willingness to track behaviors consistently, enabling more accurate self-observation, improved scheduling, and reduced procrastination.

In addition to individual behavioral gains, the intervention facilitated meaningful metacognitive development. Students used personalized weekly feedback reports and comparisons with their past records to identify inefficient patterns and set actionable goals. Unlike collaborative interventions where group processes dominate (Bhute et al., 2022), individual comparison proved more effective in this context, supporting a personalized pathway for strategy refinement. The WeChat-based technological tools strengthened this process by providing immediate visualizations of task patterns, making abstract strategies tangible through data-informed reflection and behavioral validation.

Taken together, these findings indicate that improvements were produced not by isolated activities but through the interaction of the three elements outlined in experiential-learning cycles, SRL-based regulatory processes, and technology-enabled monitoring and feedback. The cyclical movement through concrete experience, reflection, conceptualization, and active experimentation created recurring opportunities for cognitive dissonance, strategy visualization, and behavioral adjustment—mechanisms central to both experiential learning and SRL. Technology amplified these effects by supplying real-time behavioral evidence and closing the feedback loop. The alignment between observed outcomes, student feedback, and existing research reinforces both the validity of the design principles and the maturity of the overall pedagogical framework.

### 5.2. Implications

This study demonstrates theoretically that experiential learning and self-regulated learning (SRL) can jointly support the development of time-management competencies through iterative cycles of experience, reflection, conceptualization, and experimentation. Technology-supported self-monitoring tools further strengthen these regulatory processes while providing authentic behavioral data to assess learners’ time-use patterns. Practically, the six validated design principles (D1–D6) offer actionable guidance for integrating time-management instruction into university courses, including contextualized tasks, structured reflective scaffolds, technology-enabled feedback, and data-driven iterative practice. The successful incorporation of the WeChat mini-program illustrates a scalable and low-cost approach that delivers ongoing feedback without increasing instructional burden. Overall, the integrated framework and design principles provide a theoretically grounded and practically feasible model for supporting students’ development of sustainable time-management competencies.

### 5.3. Limitations and Future Directions

Several limitations of the present study should be acknowledged. First, the study was conducted within a single institution, which may limit the generalizability of the findings. Second, although the behavioral logs were generated through a WeChat-based tool, reliance on a single digital platform may introduce contextual constraints related to students’ technology familiarity and usage habits. Third, while daily self-evaluation comments provided valuable insights into students’ experiences, they may still involve elements of self-report bias and lack the precision of standardized measurement tools. Finally, each design iteration lasted only one academic semester, restricting the assessment of longer-term developmental patterns. Future research could address these issues through multi-institutional studies, extended longitudinal designs, the use of diversified digital platforms, and the incorporation of richer digital trace data.

## 6. Conclusions

This study demonstrates that an experiential learning approach, developed and refined through three iterative design cycles, effectively enhances time management competencies among first-year university students. Guided by six empirically derived design principles and supported by a dedicated digital tool, the pedagogical framework was associated with observable improvements in student outcomes. Participants in the final iteration demonstrated higher levels of their planning and task completion rates, showed more balanced time allocation, and reported higher satisfaction. These results confirm the maturity and efficacy of the framework and its supporting tools. This study offers a model that is both theoretically grounded and practically feasible for supporting students’ transition into higher education.

## Figures and Tables

**Table 1:** Instructional Design for Experiential Learning.

Theoretical Dimension	Design Principles	Design Decisions	Supporting Literature
Concrete Experience	D1. Guide students through the task-driven time management experience	Enforce students’ time management by assigning tasks that integrate the tasks into their daily lives as much as possible	(Joplin, 1981; Qudrat-Ullah, 2010)
	D2. Emphasis on the time management experience of real individuals	Emphasize the real individual experience so students can record, analyze, reflect, and adjust based on their own personal lives	(Illich, 1971; Kwon, 2021)
Reflective Observation	D3. Provide adequate scaffolding for recording and monitoring of time and tasks	Provide students with templates and framework guides for planning, documenting, and reflecting in the context of research as well as for real-world problems	(Cathro, 2020)
	D4. Support student reflection through recorded reflections and tutor guidance	Help individual students to reflect based on real-world feedback; the instructor provides guidance and feedback before the second session of the assignment based on specific examples of students’ out-of-class time management performance and the class activities	(Black et al., 2021; Steinert et al., 2016)
Abstract Conceptualization	D5. Provide adequate opportunities for group debriefing and summarization of time management situations	Provide voluntary or mandatory ways to guide students to conduct in-class debriefing presentations and summary reflections to share with their classmates during stage time	(Chen et al., 2020; Park et al., 2020)
Active Experimentation	D6. Provide adequate opportunities for students to apply what they learn in time management practice	Provide students with the opportunity to practice in two stages: the first stage is for free exploration stage, after reflecting and summarizing; in the second stage, student learning and feelings are applied again in practice	(Fromm et al., 2021; Leal-Rodríguez & Albort-Morant, 2019)

**Table 2:** Key Changes to Scaffolding Tools Across Three Iterations.

Iteration	Data Collection Format	Tool	Key Characteristics	Data Type
1st iteration	Spreadsheet-based record	Excel	Manual daily entry; end-of-day self-report	Daily self-reported duration and ratings
2nd iteration	Online daily questionnaire	Sojump	Structured daily self-report; fixed items	Daily self-reported ordinal ratings
3rd iteration	Mobile daily check-in tool	Mini-program	Mobile-based daily self-report; real-time entry and feedback	Daily self-reported records and ratings

**Table 3:** Key Changes in Student Time Management Competencies in Three Iterations.

Grade		First Iteration	Second Iteration	Third Iteration	H	*p*	*η* ^2^
Days of recording	Median	19	12	28	124.564	0.000	0.447
	Mean	18	16	27			
Total planned	Median	38	17	58	55.676	0.000	0.215
	Mean	42	21	68			
Total completed	Median	32	19	66	752.587	0.000	0.207
	Mean	40	22	69			
Completed category count	Median	5	6	6	4.659	0.097	0.021
	Mean	5	6	6			
Planned completion rate	Median	0.82	0.81	0.73	14.540	0.000	0.059
	Mean	0.88	0.82	0.66			
Daily independent learning time (min)	Median	207.17	81.85	103.35	70.705	0.000	0.269
	Mean	224.48	95.84	126.43			
Daily extracurricular time (min)	Median	40.60	52.37	88.50	14.497	0.000	0.139
	Mean	54.93	57.08	140.48			
Daily self-evaluation scores	Median	2.21	3.92	3.63	100.211	0.000	0.421
	Mean	2.34	3.85	3.53			

Note: Days of recording refers to the number of days students submitted valid daily records; Total planned is the total number of tasks planned during the recording period; Total completed indicates the number of tasks completed, regardless of whether they were originally planned or unplanned; Completed category count refers to the number of different task types completed (e.g., coursework, reading); Planned completion rate is rated on a five-point scale representing 20%–100% daily task completion; Daily independent learning time is the average time spent per day on academic activities(e.g., Coursework, Professional Practice); Daily extracurricular time refers to time spent on non-academic activities (e.g., Physical Exercise, Hobbies and Interests); Daily self-evaluation scores is a 1–5 self-rating of time management, recorded each day.

**Table 4:** Overview of Measurement Instruments and Variables in the First Iteration.

Category	Measured Variables	Instruments
Classroom observation (video and field notes)	Participation in experiential learning activities; Reported time management situation; Teacher feedback	rear-mounted cameras in the smart classroom and researchers
Student daily records	Task planning; Task execution; Self-assessment	scaffolds embedded in experiential learning activities (Excel)
Semi-structured interviews	Learning experience; Perceived changes in time management ability	Interview outline in the Supplementary Materials
Baseline questionnaire	Demographics; Prior time management experience	Questionnaire in the Supplementary Materials

**Table 5:** Evaluation of Instructional Design Principles for the First Iteration.

Dimension	Design Principles	Pros	Cons
Concrete Experience	D1.Guide students through the task-driven time management experience	a. External motivation for change b. Increase social presence	a. Awaken negative emotions
	D2. Emphasis on the time management experience of real individuals	a. Enhance personal engagement b. Integrate theory with practice c. Clear self-awareness	a. Privacy disclosure
Reflective Observation	D3. Provide adequate scaffolding for recording and monitoring of time and tasks	a. Guide recording and monitoring b. Facilitate reflection and adjustment c. Quantify issues via time data d. Clear self-awareness e. Negative mood improvement	a. Time-consuming and burdensome b. Repetitive and tedious recording c. Privacy disclosure
	D4. Support student reflection through recorded reflections and tutor guidance	a. Inspire introspection b. Method guidance c. Ameliorate learned helplessness	a. Much labor, little gain b. Delaying feedback c. Lacking targeted guidance
Abstract Conceptualization	D5. Provide adequate opportunities for group debriefing and summarization of time management situations	a. Enhance decision-making quality b. Mutual supervision	a. Peer inactivity reduces effort b. Privacy leakage
Active Experimentation	D6. Provide adequate opportunities for students to apply what they learn in time management practice	a. Mitigate reluctance to mandatory tasks b. bolster intrinsic motivation c. Enhancing Self-Perception of Time	a. Limited impact on change

**Table 6:** Evaluation of Instructional Design for the Second Iteration.

Design Principles	Major Revision in Design Decisions	Pros	Cons
D3. Provide appropriate online scaffolding for time recording and monitoring	Sojump (online link) to fill in	a. Simple and quick b. Balancing privacy and transparency c. Charting the path to improvement d. Reflecting on plans and contingencies e. Fostering emotional literacy	a. Personalization constrained b. Inconvenient to click and save c. Lost online inputs
D4. Support student reflection through feedback and tutor guidance	Data analysis table (teacher assistant)	a. Inspire introspection b. Method guidance c. Ameliorate learned helplessness	a. Much labor, little gain b. Delaying feedback c. Lacking targeted guidance
D5. Provide data support and autonomy for team summarization and reporting	Summary based on data discussion	a. Enhance decision-making quality b. Mutual supervision	a. Peer inactivity reduces effort b. Privacy leakage

**Table 7:** Modified Instructional Design Principles for Experiential Learning.

Theoretical Dimension	Design Principles	Design Decisions
Concrete Experience	D1. Guide students through the task-driven time management experience	Enforce students’ management of their time by assigning tasks that they can integrate into their daily lives as much as possible
	D2. Emphasis on the time management experience of real individuals	Emphasize real individual experience, so students can manage their time based on their own personal lives and habits.
Reflective Observation	D3. Provide a task-categorization scaffold that enables students to plan, monitor, and evaluate their tasks systematically	Provide students with portable, instructional, privacy-sensitive formwork, and scaffolding
	D4. Support student reflection through visual feedback and tutor guidance	Feedback-guided reflection based on real data, automated feedback from teachers, and technology
Abstract Conceptualization	D5. Comparative analysis of individual situations and planning for improvement	Provide voluntary or mandatory means for students to report and reflect on their personal time management and share it with their classmates
Active Experimentation	D6. Provide adequate opportunities for students to apply what they learn in time management practice	Provide students with the opportunity to practice in two stages: the first stage is for free exploration; after reflecting and summarizing, students’ learning and feelings are applied again in practice in the second stage

## Data Availability

The data used to support the findings of this study are available at Mendeley Data (https://data.mendeley.com/preview/dy8ycy7rby?a=7629ab1f-5473-4914-989d-5193cc71e769, accessed on 29 September 2025; https://data.mendeley.com/preview/jb96ynp79z?a=5a1d230d-ea16-40eb-90a4-f9cf0d7b6410, accessed on 17 December 2025).

## Supplementary Materials

The following supporting information can be downloaded at: https://www.mdpi.com/article/10.3390/bs16010027/s1.
