# Supplementary material for: Developing Time Management Competencies for First-Year College Students Through Experiential Learning: Design-Based Research
Source: Behav Sci (Basel). 2025 Dec 22;16(1):27. doi: 10.3390/bs16010027 (PMC12837181; doi:10.3390/bs16010027)
Supplement: Supplementary file 1 [file behavsci-16-00027-s001.zip › behavsci-3946287-supplementary.pdf]

Supplementary Materials

Supplementary Materials .... **Error! Bookmark not defined.**
Supplemental S1. Instruments 2
S1.1. Questionnaire 2
S1.2. Interview Outline (Semi-Structured) 2
S1.3. Daily Behavior Record Template 3
S1.4. Data Collection Interfaces (Implementation Examples) 4
Supplemental S2. Data Extraction and Variable Definitions 5
S2.1. Variables from the Questionnaire 5
S2.2. Variables from Daily Behavior Records 5
S2.3. Relation to Main Outcome Constructs 6
Supplemental S3. Course Design and Experiential Learning Cycles 7

Supplemental S1. Instruments

S1.1. Questionnaire

- Purpose: The questionnaire collected students' basic information and prior time management experience. The data were used to describe the sample and provide background for later analysis.
- Timing: The questionnaire was distributed at the beginning of each iteration, before any instructional activities or intervention tasks were introduced.

1. Gender: ☐ Male ☐ Female   2. Age: _____ years
3. Academic major: ________
4. Before this course, did you have time management Experience?

   ☐ No experience at all.   ☐ I've heard of it, but I've never done it myself.

   ☐ Tried but didn't stick to it.   ☐ Have your own personalized experience.

   ☐ Other________________________

S1.2. Interview Outline (Semi-Structured)

- Purpose: The interviews explored students' evolving perceptions of time management competencies, learning experiences, and behavioral adaptation processes across three iterative phases.
- Timing: The interviews were conducted after the completion of each iteration, once all tasks and recordings within that iteration had ended.

Section 1: General Learning Experience

- How would you describe your overall learning experience in this course?
- What aspects of the course design stood out to you as particularly impactful?

Section 2: Time Management Competency Development

- How did you adapt your daily or weekly routines based on the course tasks and your own preferences or working habits?
- How has your understanding of time management evolved during the course?
- Can you describe a specific instance where your time management strategy succeeded or failed? What did you learn?
- What external or internal factors most significantly influenced your ability to manage time effectively?

Section 3: Impact of Recording & Reflection Tools

- How did the daily recording process affect your awareness of time allocation?
- Did you feel that the time management strategies you used were tailored to your personal style or needs? Why or why not?
- In what ways did the reflection prompts shape your approach to planning and self-evaluation?
- How effective do you think recording tools are for you? What are their advantages and disadvantages? And why?

Section 4: Collaborative Learning Dynamics

- How did group discussions or peer interactions influence your time management decisions?
- Can you share an example of how collaborative feedback altered your weekly planning?

Section 5: Adaptive Feedback & Iterative Design

- What changes to the recording tools or feedback mechanisms would enhance their utility for future cohorts?
- If you could redesign one element of this course to better support time management skill development, what would it be?

S1.3. Daily Behavior Record Template

- Purpose: This record was used to track students' daily planning, task completion, time use, and self-evaluation during the course. It also provided the daily learning experiences and basic reflective information that supported the experiential learning cycle, forming the initial data from which students later conducted deeper reflection and strategy adjustment. Across all three iterations, the same data fields in this template served as the basis for analyzing students' time management performance. The specific interfaces or digital versions used in each iteration correspond to Figures 2, 3, and 4 in the main text.
- Timing: Students completed the daily record throughout each iteration, as part of the regular learning and self-monitoring activities.

Table S1. Daily Behavior Record

Recording Table for Daily Behaviors<br>Student ID: __________ Name: __________ Date (Month/ Day/ Year)					
**Sleep**	Last night's bedtime		__________:__________		
	Today's wake-up		__________:__________		
	Last night's sleep quality		①②③④⑤		
**Classroom learning**	Learning Engagement		Active Participation		
	①②③④⑤		①②③④⑤		
	Eliminating Distraction		Learning Effectiveness		
	①②③④⑤		①②③④⑤		
**With or without a plan**	**Completion Rate**	**Duration**	**Self-Evaluation**		
○Coursework	20% 40% 60% 80% 100%	____min	Concentration Level<br>①②③④⑤	Efficiency<br>①②③④⑤	Quality of Completion<br>①②③④⑤
○Additional Learning	20% 40% 60% 80% 100%	____min	Objective and Plan Progress<br>①②③④⑤	Proactiveness<br>①②③④⑤	Quality of Study<br>①②③④⑤
○Professional Practice	20% 40% 60% 80% 100%	____min	Readiness<br>①②③④⑤	Skill Proficiency<br>①②③④⑤	Practice Effectiveness<br>①②③④⑤
○Reading	20% 40% 60% 80% 100%	____min	Habit Formation<br>①②③④⑤	Open Themes<br>①②③④⑤	Reading Effectiveness<br>①②③④⑤
○Physical Exercise	20% 40% 60% 80% 100%	____min	Exercise Motivation<br>①②③④⑤	Exercise Experience<br>①②③④⑤	Exercise Effectiveness<br>①②③④⑤
○Hobbies and Interests	20% 40% 60% 80% 100%	____min	Persistence<br>①②③④⑤	Experience and Feelings<br>①②③④⑤	Skills Improvement<br>①②③④⑤
○Social Activities	20% 40% 60% 80% 100%	____min	Social Initiative<br>①②③④⑤	Physical and Mental Adjustment<br>①②③④⑤	Time Management<br>①②③④⑤
○Electronics	20% 40% 60% 80% 100%	____min	Spend____ minutes on learning	Spend____ minutes on entertainment	Self-Discipline Status<br>①②③④⑤
**Today's Reflection:**					
**Self-Evaluation Score:** ①②③④⑤				**Mood Score:** ①②③④⑤	

S1.4. Data Collection Interfaces (Implementation Examples)

This section provides representative interface-level examples illustrating how the daily behavior record was implemented across different data collection formats in the three iterations. These examples are intended to demonstrate the practical implementation of the same set of behavioral indicators, rather than to introduce additional measures or analytic variables. All core indicators and variable definitions are specified in Appendix A.3 and Appendix B.

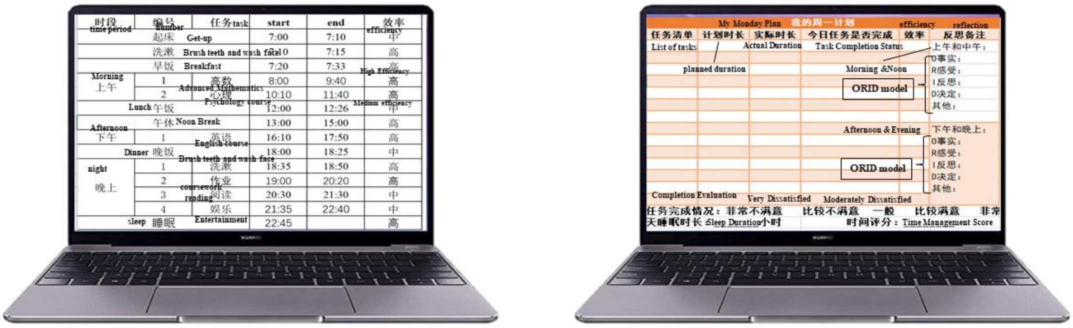

Figure S1. Spreadsheet-Based Daily Record (First Iteration)

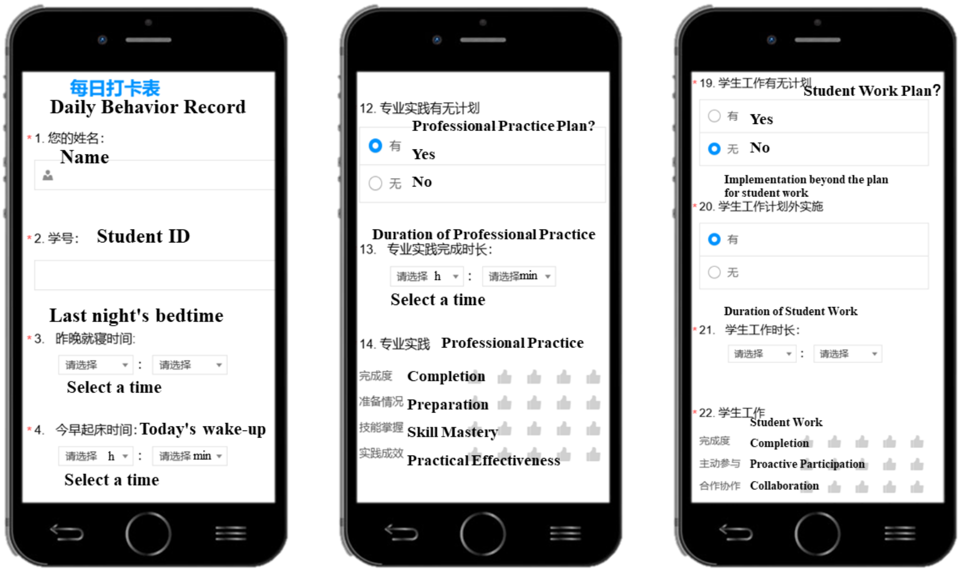

Figure S2. Questionnaire-Based Daily Record (Second Iteration)

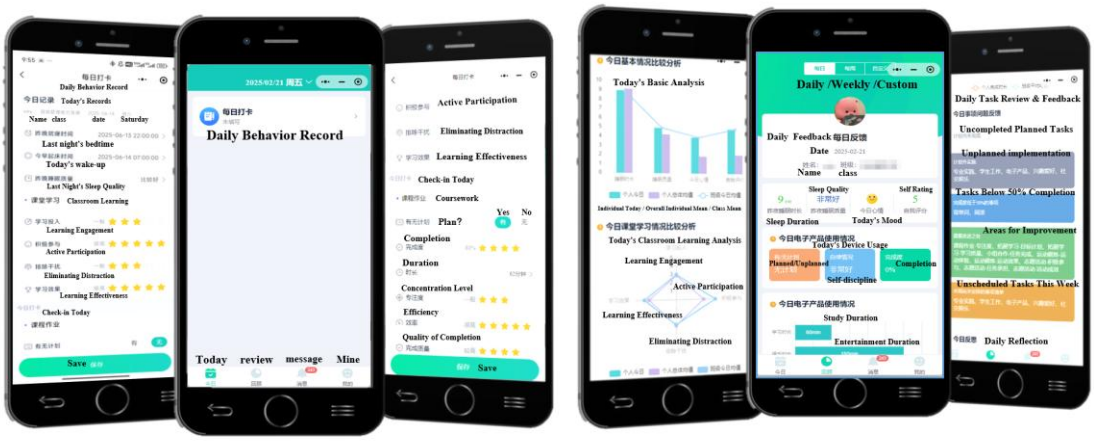

(a) **(b)**

Figure S3. Mobile Mini-Program Interface (Third Iteration): **(a)** Data entry interface of the WeChat mini-program for daily check-ins; **(b)** corresponding visualization feedback page.

Supplemental S2. Data Extraction and Variable Definitions

S2.1. Variables from the Questionnaire

From the baseline questionnaire(Appendix A.1), the following information was extracted for descriptive and grouping analyses:

- Demographic variables: gender, age, academic major
- Prior time management experience: categorized into four levels
  (1) No experience at all
  (2) Heard of it but never practiced
  (3) Tried but discontinued
  (4) Have sustained personal experience

These variables were used solely for sample description. In combination with interview data and behavioral logs, they served to analyze students' perceptions of experiential learning and their attitudes toward the intervention. No composite scale was constructed from the questionnaire.

S2.2. Variables from Daily Behavior Records

Based on the Daily Behavior Record (Appendix A.2), the following variables were extracted and computed directly from students' daily submissions using fixed rules across all three iterations, with original field names shown in *italics*.

- Days of recording: the total number of days for which the student submitted a valid daily behavior record.
- Total planned: the sum of all tasks marked as *With a Plan* during each iteration.
- Total completed: the sum of all tasks whose *Completion Rate* was greater than 0 during each iteration.
- Completed category count: the number of task categories in which the student recorded a *Completion Rate* greater than 0 during the iteration.
- Planned completion rate: the average *Completion Rate* of all tasks marked as *With a Plan*.
- Daily independent learning time (min): the daily sum of *Duration* across academic-related categories (*Coursework, Additional Learning, Professional Practice, Reading, Electronics–learning*).
- Daily extracurricular time (min): the daily sum of *Duration* across non-academic categories (*Physical Exercise, Hobbies and Interests, Social Activities, Electronics–entertainment*).
- Daily self-evaluation scores: the student's recorded *Self-Evaluation Score* for that day.

All variables were based on students' self-reported daily submissions. Instead of using a standardized time management scale, we applied a process-based behavioral logging approach to track changes in planning, execution, and reflection over time. Some indicators were recorded as ordinal ratings for descriptive and comparative purposes only.

S2.3. Relation to Main Outcome Constructs

The indicators above were used to examine four key aspects of students' time management competences discussed in the main text. Each aspect corresponds to a different stage of the self-regulation process, and each one is reflected through specific behaviors captured in the daily records. These measures together provide a fuller picture of how students planned their work, carried out tasks, managed their time, and reflected on their progress across the course.

- Planning ability (Planning Stage): reflected by total planned tasks and planned completion rate.
- Execution and Monitoring (Performance Stage): reflected by total completed tasks and completed category count, indicating how students carried out planned tasks and monitored their progress.
- Time allocation (Performance Stage): reflected by daily independent learning time and daily extracurricular time, showing how students allocated and regulated their time and learning resources.
- Self-Reflection (Reflection Stage): reflected by the daily self-evaluation score and interview responses, indicating how students assessed their performance and adjusted strategies.

Looking at several types of indicators allowed us to understand students' learning habits from multiple angles rather than relying on a single measure. In addition to the daily logs, we also drew on interview responses to better understand students' own interpretations of their behaviors. Comparing what students recorded each day with what they later described in interviews helped us confirm patterns, clarify inconsistencies, and ground our interpretations in both behavioral and self-reported evidence. This combined approach increases the clarity of the measurement process and supports the trustworthiness of the findings.

Supplemental S3. Course Design and Experiential Learning Cycles

The 12-session course followed a cyclical experiential learning (EL) structure, organized into one large cycle and four embedded mini-cycles. Every two sessions formed a repeated pattern in which students first engaged in a new learning experience, recorded and reflected on their behaviors after class, and then received conceptual guidance in the following session. This structure allowed students to move continuously through experience, reflection, conceptualization, and application while gradually strengthening their time management (TM) competences. The table below outlines the focus and activities of each session within this design.

Table S2. Overview of the 12-Session Course Design

Stage of Large Cycle	Stage of Mini-Cycles	Session	Focus	Activities & Learning Process	Outcome
Concrete Experience & Reflection Observation	CE → RO	1	Entering EL	**CE:** Teacher introduces TM and tasks.<br>**RO:** Students reflect on initial habits during class discussion.	Initial awareness of TM and daily behaviors
	AC → AE	2	Reflection & Concept Building	**AC:** Teacher explains basic SRL/time-use concepts.<br>**AE:** Students apply concepts in daily recording after class.	Recognize patterns and try applying early concepts
	CE → RO	3	Planning Experience	**CE:** New planning and monitoring tasks assigned.<br>**RO:** Students review and discuss task experience in class.	Experience applying planning tasks
	AC → AE	4	Reflection & First Report	**AC:** Teacher offers conceptual guidance for improvement.<br>**AE:** Students complete SRL Report 1.	Produce first reflective report and refine strategies
Abstract Conceptualization		5	Planning	Introduce principles of planning, goal-setting, and prioritization; explain how to construct daily and weekly plans.	Conduct reflective summaries, extract lessons learned, and articulate strategy adjustments for the next cycle.
		6	Execution and Monitoring	Demonstrate how to use structured tools to track tasks, monitor progress, and evaluate effectiveness.	
		7	Time allocation	Teach methods for categorizing activities, balancing academic and non-academic tasks, and interpreting time-use data.	
		8	Reflection	Guide students in interpreting learning data, connecting experiences with conceptual	

				understanding, and forming improvement strategies.	
Active Experimentation	CE → RO	9	Mini - Program Experience	**CE:** Students use the mini-program for daily tracking. **RO:** Class reflection on early use and feedback.	Experience automated feedback in daily practice
	AC → AE	10	Reflection with Visual Data	**AC:** Teacher explains visual data patterns. **AE:** Students apply understanding to adjust routines.	Reflect using visual trends and adjust behaviors
	CE → RO	11	Strategy Refinement	**CE:** Students test refined strategies in real contexts. **RO:** Class reflection on improvements and challenges.	Strengthen long-term SRL strategy use
	AC → AE	12	Final Reflection & Report	**AC:** Teacher leads final synthesis. **AE:** Students prepare SRL Report 3 and future plan.	Summarize learning and plan ongoing adjustment
